# Chitin and chitin-related compounds in plant–fungal interactions

**DOI:** 10.1080/21501203.2018.1473299

**Published:** 2018-05-15

**Authors:** Tünde Pusztahelyi

**Affiliations:** Central Laboratory of Agricultural and Food Products, Faculty of Agricultural and Food Sciences and Environmental Management, University of Debrecen, Hungary

**Keywords:** Chitin, chitosan, chitinase, plant–fungal interaction, PR protein

## Abstract

Chitin is the second abundant polysaccharide in the world after cellulose. It is a vital structural component of the fungal cell wall but not for plants. In plants, fungi are recognised through the perception of conserved microbe-associated molecular patterns (MAMPs) to induce MAMP-triggered immunity (MTI). Chitin polymers and their modified form, chitosan, induce host defence responses in both monocotyledons and dicotyledons. The plants’ response to chitin, chitosan, and derived oligosaccharides depends on the acetylation degree of these compounds which indicates possible biocontrol regulation of plant immune system. There has also been a considerable amount of recent research aimed at elucidating the roles of chitin hydrolases in fungi and plants as chitinase production in plants is not considered solely as an antifungal resistance mechanism. We discuss the importance of chitin forms and chitinases in the plant–fungal interactions and their role in persistent and possible biocontrol.

**Abbreviations** ET, ethylene; GAP, GTPase-activating protein; GEF, GDP/GTP exchange factor; JA, jasmonic acid; LysM, lysin motif; MAMP, microbe-associated molecular pattern; MTI, MAMP-triggered immunity; NBS, nucleotide-binding site; NBS-LRR, nucleotide-binding site leucine-rich repeats; PM, powdery mildew; PR, pathogenesis-related; RBOH, respiratory burst oxidase homolog; RLK, receptor-like kinase; RLP, receptor-like protein; SA, salicylic acid; TF, transcription factor.

## Introduction

Chitin is the second abundant polysaccharide (N-acetyl-glucosamine polymer) on Earth. In nature, chitin can be found in a different form in crabs, insects or fungi. Microscopically, it is in a crystalline or semi-crystalline form (Pillai et al. 2009), which makes this polysaccharide into a rigid and resistant material that very powerful working as a barrier in cell wall or cuticle and protects the organisms itself. This feature is entire of another essential polysaccharide: cellulose, the structural homolog of chitin.

**Figure 1.**
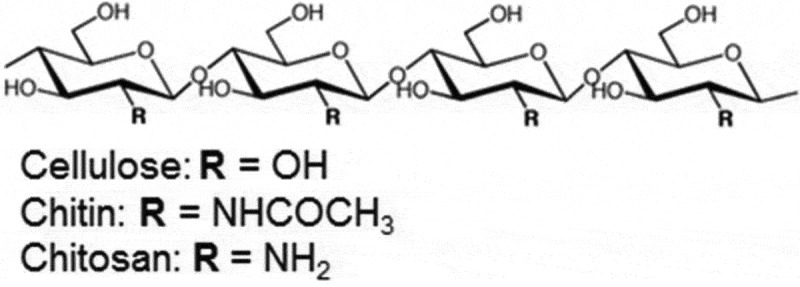
Structural homology of linear polysaccharides. Chitin, chitosan and cellulose (Azuma et al. 2015).

In organisms, other than the above-mentioned, chitin polymers and its modified form, chitosan [β-(1→4)-linked D-glucosamine (deacetylated unit) and N-acetyl-D-glucosamine (acetylated unit)] (Figure 1) are rare. Compared to chitin, chitosan is unique and found only in fungi that have deacetylase enzymes.

Chitin and chitosan induce host defence responses in both monocotyledons and dicotyledons. These reactions include the following: ion flux variations, cytoplasmic acidification, membrane depolarisation and protein phosphorylation (Felix et al. 1993, 1998), chitinase and glucanase activation (Roby et al. 1987; Tayeh et al. 2015), lignification (Kawasaki et al. 2006; Ali et al. 2014), generation of reactive oxygen species (ROS) (Kuchitsu et al. 1995), biosynthesis of jasmonic acid (JA) (Nojiri et al. 1996), and phytoalexins (Ren and West 1992; Yamada et al. 1993), and the expression of early responsive and defence-related genes (Minami et al. 1996; Libault et al. 2007). Moreover, chitosan induces proteinase inhibitors (Walker-Simmons and Ryan 1984), phytoalexin biosynthesis (Hadwiger and Beckman 1980) and callose formation (Köhle et al. 1985) in dicot species. The plants’ response to chitin, chitosan and the derived oligosaccharides depends on the acetylation degree (Akiyama et al. 1995; Cord-Landwehr et al. 2016; Li et al. 2016) and the degree of polymerisation (Walker-Simmons and Ryan 1984; Li et al. 2016).of these compounds.

Chitinases are involved in the early events of host–parasite interactions of biotrophic and necrotrophic mycoparasites and entomopathogenic fungi, and vesicular-arbuscular mycorrhizal fungi (Sahai and Manocha 1993). Here, we discuss the importance of chitin forms and chitinases in the plant–fungal interaction and biocontrol.

## PR proteins in plants

In plants, inducible defence-related proteins have been described upon infection with fungi, bacteria, viruses, oomycetes, or insect attack. Pathogenesis-related (PR) proteins are plant proteins that are induced in response to pathogen attack and have been classified into 17 families (Van Loon et al. 2006). These proteins are grouped according to their sequence similarity and biological activity. PR-2 is a β-1,3-glucanase; PR-3, PR-4, PR-8 and PR-11 are different types of chitinase; PR-5 is a thaumatin-like protein; PR-6 is a proteinase inhibitor; PR-7 is an endoproteinase; PR-9 is a peroxidase; PR-10 is a ribonuclease; and PR-12, PR-13 and PR-14 are a defensin, thionin and lipid-transfer proteins, respectively (reviewed by Van Loon and Van Strien 1999). Chitinolytic enzymes (chitinase or endo-β-N-acetylglucosaminidase; EC 3.2.1.14 and exochitinases, or β-N-acetylhexosaminidase; EC. 3.2.1.52) belong to the above-mentioned four recognised families of PR proteins (PR-3, PR-4, PR-8 and PR-11) that respond to attack by potential pathogens. The PR-4 family comprises class-I and class-II chitinases (Bravo et al. 2003). Moreover, abiotic stresses like UV-C (El Ghaouth et al. 2003), CO_2_ (Goñi et al. 2009), ROS (Kumar et al. 2009) and a variety of other treatments also elicit the plant response (Bravo et al. 2003; Van Loon et al. 2006).

In a plant genome, several chitinases are encoded. For example, Hawkins et al. (2015) characterised 33 chitinase genes in maize (*Zea mays*) genome via sequence diversity and expression patterns. The recent evolution of this gene family was also noted in their work. Seven chitinase genes were identified that had alleles associated with increased resistance to aflatoxin accumulation and *Aspergillus flavus* infection in a field trial. The expression patterns of these genes support possible grain resistance mechanisms. PR4 (class-II chitinase) mRNA accumulation was stimulated by treatment with silver nitrate in maize, whereas the application of the hormones gibberellic acid or acetylsalicylic acid did not affect. Wounding, or treatment with abscisic acid (ABA) or methyl jasmonate, resulted in the accumulation of ZmPR4 mRNA in maize leaves (Bravo et al. 2003).

The differential expression of the entire set of chitinolytic enzymes in maize in the presence and absence of a pathogenic fungus, e.g. *Trichoderma harzianum* was also examined (Shoresh and Harman 2010). Expression of the chitinolytic enzymes revealed a protein band with chitinase activity after gel electrophoresis that had higher mass than any known chitinase. Shoresh and Harman (2010) reported the characterisation of this new protein, a heterodimer between an exo- and an endo-enzyme. The endo portion differed between plants colonised with *T. harzianum* and those grown in its absence and between shoots and roots. The dimeric enzyme from the *Trichoderma*-inoculated plants had higher specific activity and more excellent ability to inhibit fungal growth than those from control plants. Moreover, the activity of specific chitinolytic enzymes was higher in plants grown from *Trichoderma*-treated seeds than in control plants. As it was shown, plant chitinase expression can be tissue specific. Campos-Bermudez et al. (2013) also observed differential expression of chitinase in silk and kernels of maize, indicating that some gene is kernel specific, or at least is not expressed in silks.

Plant hormones induce stress resistance and endogenous signalling molecules in plants, e.g. ethylene (ET) (Ton et al. 2002), salicylic acid (SA) (Janda and Ruelland 2014), JA (Wasternack 2007; Van Der Ent et al. 2009) and ABA (Hauser et al. 2011) have been associated with plant defence signalling against biotic stress. SA signalling induces protection against biotrophic pathogens, whereas JA against necrotrophic pathogens (Glazebrook 2005). It was found that SA and JA induction initiated a different expression level of chitinases in plants. For example, in *Brassica juncea*, Rawat et al. (2017) characterised a class-IV chitinase gene (*BjChp*) in response to fungal infection, SA and JA treatments and wounding. Gene expression studies revealed that the transcript levels of *BjChp* gene increased significantly both in local and distal tissues after *Alternaria brassicae* infection. *BjChp* gene was induced by JA and wounding but moderately by SA (Rawat et al. 2017).

Seemingly, chitinase expression is not depended on the resistance of the cultivar. Ha et al. (2016) analysed eight wheat genes, and seven were more strongly induced by *Fusarium graminearum* (Fusarium head blight) than by Magnaporthe wheat blast. Genes for chitinase (Chi2), β-1,3-glucanase (PR2), a plant defensin homolog (PRPI), and a peroxidase (Pox2) were strongly upregulated in response to both pathogens independently to the resistance phenotypes of the cultivar against the pathogen. Meanwhile, PR2 and PR5 (thaumatin-like) proteins were only transiently triggered by the wheat blast (Ha et al. 2016).

## Resistance and signalling in plants

In plants, resistance genes (R-genes) act as an immune system by recognising pathogens and inducing defensive pathways. Many R-gene loci are present in plant genomes, presumably reflecting the need to maintain a vast repertoire of resistance alleles (*e.g*. Fekete et al. 2009). In addition to the R gene-mediated pathways of plant resistance to specific pathogens, plants can recognise several microbial surface-derived molecules, which elicit a general immune response in both host and non-host plants. Plants distinguish microbes via perception of conserved microbe-associated molecular patterns (MAMPs) to induce MTI, which is sufficient to restrict microbial growth and eventually leads to cell death (Eckardt 2008). These conserved MAMPs include peptidoglycans from Gram-positive bacteria, lipopolysaccharides of Gram-negative bacteria, or eubacterial flagellin. Glucans, chitins and proteins originate from fungal cell walls (Nürnberger and Brunner 2002) from which chitin does not compose plant cells and, therefore, represents an ideal MAMP. Plant secreted chitinolytic enzymes hydrolyse fungal cell wall that results in loss of cell integrity and results in releasing the small chitin fragments. Chito-oligosaccharides in their unmodified form with a degree of polymerisation of 6 to 8 are strong inducers, which efficiently trigger MTI. Chitin oligomers are released during pathogen ingress and are recognised by plants. However, the actual length and concentration as well as the amount of the oligosaccharides that is needed to induce an exact immune response in plants that are released at infection sites have remained to be elucidated (Sánchez-Vallet et al. 2015).

Investigation of the molecular mechanism of chitin perception and chitin-triggered immunity in plants flourished since the cloning of the first plant chitin oligosaccharide receptor CEBiP (chitin elicitor-binding protein), a plasma membrane glycoprotein from rice (Kaku et al. 2006). CEBiP is typical receptor-like protein (RLP) with an extracellular domain containing two predicted lysin motifs (LysMs) at the N terminus and a short membrane-spanning domain at the C terminus and lacks cytoplasmic kinase domain but directly involved in chitin-triggered immunity (Kouzai et al. 2014).

In the *Arabidopsis* genome, five chitin elicitor LysM receptor-like kinase 1 (LysM RLKs) are encoded, of which only one was implicated in chitin detection and named AtCERK1 for chitin elicitor receptor kinase 1 or LysM RLK1 (Miya et al. 2007; Wan et al. 2008). Structurally, AtCERK1, an RLK, is composed of a transmembrane domain, three tandem LysMs in its ectodomain and an intracellular serine/threonine kinase domain (Miya et al. 2007; Wan et al. 2008) in *Arabidopsis*. Chitin-induced AtCERK1 homodimerisation has been proved to be necessary for its activation (Liu et al. 2012). The chitin-induced signalling in *Arabidopsis* also required two other LysM-RLKs, named AtLYK4 and AtLYK5 (Wan et al. 2012; Cao et al. 2014). AtLYK5 shared overlapping function with AtLYK4 and interacted with AtCERK1 in a chitin-dependent manner. Mutations in AtLYK5 resulted in a significant reduction in the plant chitin response (Cao et al. 2014).

In the situation of defence responses to pathogenic fungi, LysM RLK1 (Wan et al. 2008) or CERK1 (Miya et al. 2007)/CERK1 (as in *Arabidopsis*) or CERK1/CEBiP receptor dimers (as in rice, Shimizu et al. 2010) needs longer chito-oligomers, like octamers to activate its response through heterodimerisation (Hayafune et al. 2014; Liu et al. 2015). The perception of shorter oligosaccharides such as chitotetraose could instead rely on monomeric receptors (Miyata et al. 2014; Shinya et al. 2015) as into each receptor’s LysM domain, half of this oligomer molecule (C4) fits (Liu et al. 2012; Shinya et al. 2015).

**Figure 2.**
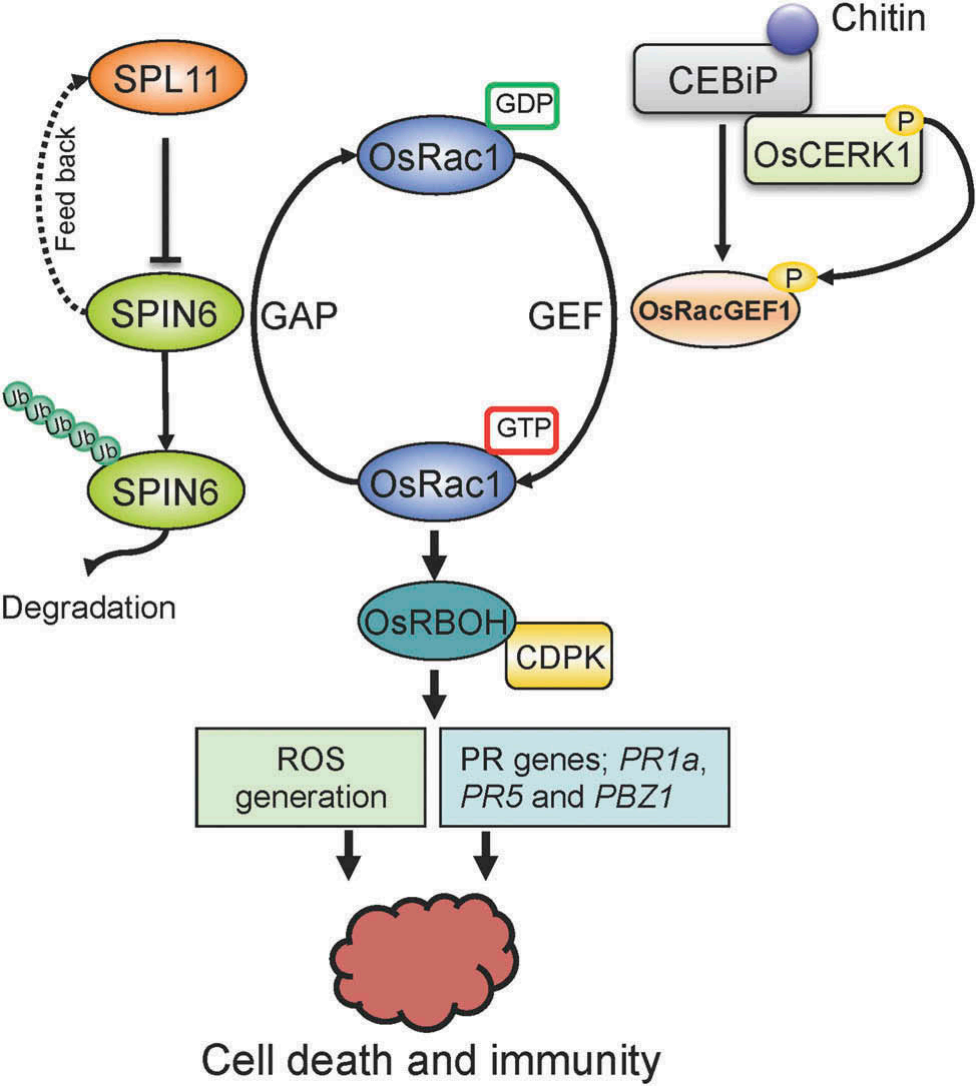
Proposed working model of the signal transduction in rice. By associating with RhoGAP protein SPIN6, the E3 ubiquitin ligase SPL11 negatively modulates OsRac1-mediated immune signalling. SPIN6 is ubiquitinated and degraded by the E3 ligase SPL11 via the 26S proteasome system. From the GDP state to the GTP state, OsRac1 is activated by the GEF protein OsRacGEF1, then associates with the NADPH oxidases OsRBOH/CDPK complex to trigger ROS generation. The activation of OsRac1 requires the phosphorylation by the kinase protein OsCERK1, a co-receptor of the MAMP effector chitin. OsCERK1 dimerises with LysM protein CEBiP1 to perceive chitin signalling. The interaction between SPIN6 and OsRac1 may lead to the change of OsRac1 from the GTP state to the GDP state, which reduces the active form of OsRac1 in rice cells. Mutation in the Spin6 gene may cause accumulation of ROS and PR proteins, such as PR1a, PR5 and PBZ1, that results in plant cell death and immunity (Liu et al. 2015).

Plant Rac/Rop small GTPases are a plant-specific Rho family of small GTPases, which are regulated by shuttling between a GDP-bound inactive form and a GTP-bound active form (Figure 2). Two regulatory factors, GDP/GTP exchange factors (GEFs) and GTPase-activating proteins (GAPs), mediate this shuttling. OsRac1 is one of the seven rice Rac/Rop GTPases and plays essential roles in chitin-induced immune responses. However, it represents a part not only in the expression of immune-related genes but also ROS production and lignification (Kawasaki et al. 1999, 2006; Ono et al. 2001; Wong et al. 2007). OsRacGEF1, the GEF for OsRac1, is a part of the plant-specific ROP-nucleotide exchanger (PRONE)-type GEF family. The cytoplasmic domain of OsCERK1 interacts with the OsRacGEF1 and phosphorylates it directly (Akamatsu et al. 2013). Therefore, another type chitin signalling pathway consisting of OsCERK1 – OsRacGEF1 – OsRac1 is also presented in rice. An OsCERK1-mediated immunity branching at OsRLCK185 and OsRacGEF1 was demonstrated (Akamatsu et al. 2013).

Under chitooctaose treatment, a total of 118 transcription factor (TF) genes and 30 ubiquitin-ligase (Duplan and Rivas 2014) genes responded in *Arabidopsis thaliana* in the Affymetrix Arabidopsis whole-genome array studies. Among these genes, members from four TF families were overrepresented. 27 APETALA2/ET-responsive element-binding proteins (Phukan et al. 2017), 14 C2H2 zinc finger proteins (McGrath et al. 2005), 11 MYB domain-containing proteins (Singh et al. 2002), and 14 WRKY domain TFs (Eulgem et al. 2000) were identified. Transcript variants of a few of these genes were found to respond differentially to chitin that suggested transcript-specific regulation of these TF genes (Libault et al. 2007).

The Mediator is a highly conserved protein complex which links DNA-bound TFs to RNA polymerase II during transcription and has a role in plant immunity against necrotrophic fungal pathogens. This protein complex exists in multiple functionally distinct forms and acts as either a transcriptional activator or a repressor, conditionally on its associated protein partners. The Mediator core contains more than 20 subunits, which are organised into the head, middle, and tail modules. Many of the *Arabidopsis* mediator (MED) subunits have been implicated in immune responses. For instance, MED14, MED15, MED16 and MED19a have been shown to regulate the SA-triggered immunity against biotrophic and hemibiotrophic pathogens (Canet et al. 2012; Zhang et al. 2012, 2013; Caillaud et al. 2013). MED8, MED12, MED13, MED14, MED16, MED21, MED25 and CDK8 have been found to function in JA/ET-mediated immunity against necrotrophic pathogens (Dhawan et al. 2009; Zhang et al. 2012). MED18 also operates in resistance to necrotrophic pathogens, but the resistance appears to be independent of the JA/ET signalling (Lai et al. 2014). Wang et al. (2016) showed that MED33A/B contributed to the necrotrophic fungal pathogen *Botrytis cinerea* induced expression of the *PDF1.2* defensin, *HEVEIN-LIKE* and *BASIC CHITINASE* defence genes and is required for full-scale basal resistance to *B. cinerea*, which demonstrated a decisive role for MED33 against necrotrophic fungal pathogens.

## Chitin derived molecules and symbiotic signalling

Chitin-derived molecules not only induce plant immune responses, but they also seem to be symbiotic signalling compounds (reviewed by Genre and Russo 2016). The LysM motif is known to exist in the putative Nod-factor receptor kinases involved in the symbiotic signalling between leguminous plants and rhizobia (Desaki et al. 2017). Chitin-oligomers from fungal pathogens are structurally like lipo-chito-oligosaccharide Nod factors produced by rhizobia, and LysM RLK1 (Wan et al. 2008) or CERK1 (Miya et al. 2007) is homologous to legume Nod factor receptors (NFR) NFR1 and NFR5 (Kaku et al. 2006).

A symbiotic model emerged where the assembly of different membrane-residing receptor complexes (Limpens et al. 2015; Shinya et al. 2015) depends on which receptors are expressed by each cell type and possibly which signalling molecule is present. From OsCERK1/CEBiP receptor dimer, knock-out mutants of OsCERK1 receptor kinase was found to be essential for chitin signalling in rice, where not only chitin-triggered defence responses but also arbuscular mycorrhizal symbiosis was impaired, indicating the bifunctionality of CERK1 in defence and symbiosis (Miyata et al. 2014). On the other hand, a knock-out mutant of CEBiP, which forms receptor complex with CERK1 and was essential for chitin-triggered immunity, established mycorrhizal symbiosis. Therefore, OsCERK1 but not chitin-triggered immunity was required for arbuscular mycorrhizal symbiosis (Miyata et al. 2014). Sánchez-Vallet et al. (2015) proposed that CERK1 has a dual function in symbiosis and immunity, and acts as an adaptor in multiple receptor complexes, rather than as a receptor for a specific ligand.

It was also concluded that various LysM domains could recognise differences in their substrates, potentially, posttranslational modifications such as glycosylation of the LysM receptors can lead to specificity in substrate recognition (Mulder et al. 2006; Chen et al. 2014). While the results indicated the involvement of partially homologous plasma membrane proteins (RLK1 and NFR) both in defence and symbiotic signalling in plant cells (Kaku et al. 2006), Bozsoki et al. (2017) revealed that distinct receptor sets respond to chitin and lipochitin oligosaccharides in *Lotus japonicus* and *Medicago truncatula* legume roots separating defence from the symbiosis of the roots.

## Fungal potential against host immune system

Many pathogens make their first contact with plant cells in the apoplast, the extracellular space in plant tissue that is also a source of nutrients and shelter for many microbes. The fungal infection induces the expression of hydrolytic enzymes that accumulates at the site of invasion in plants. For example, *Blumeria graminis f.sp. tritici*, an obligate aerial biotrophic fungus, caused PM in wheat (*Triticum aestivum*). When penetration of appressorial germ tube took place, upregulation of chitinases and PR1-encoding genes occurred along with an increase of chitinase activity (Tayeh et al. 2015). The plant hydrolytic activities establish decomposition of microbial matrices: plant chitinases and glucanases disrupt the integrity of fungal walls and release chitin and glucan to generate soluble pattern-recognition receptor ligands. Since plant chitinases directly affect fungal viability and promote fungal recognition, it can be speculated that fungal pathogens evolved diverse strategies to protect themselves against deleterious effects of chitinases. Among several approaches that developed in plant pathogens to prevent recognition and MAMP-triggered activation of immune responses, alterations in the composition and structure of cell walls, modification of carbohydrate chains and secretion of effectors to protect the cell wall or to target host immune responses are well-known.

The necrotrophic plant pathogens *Botrytis cinerea, Sclerotinia sclerotiorum, Sclerotinia minor* and *Sclerotium rolfsii* can degrade or sequester two widespread plant PR proteins: a type-IV plant chitinase and a thaumatin-like protein. In comparison to protease activity, the sequestering capacity of the fungal glucan matrix seems to play a more significant role in the fungal defence against the plant thaumatin-like protein and chitinase (Marcato et al. 2017).

Conversion of chitin to chitosan by deacetylation (Cord-Landwehr et al. 2016) in host invasion may protect hyphae of pathogenic fungi from being hydrolysed by extracellular plant chitinases, as chitosan is a poor substrate for chitinases, and consequently reduce the release of elicitors (Ride and Barber 1990).

Several fungal pathogens are known to secrete effectors that can shield fungal hyphae and thus prevent access of chitinases to the chitin in the cell wall. For example, cerato-platanins are small secreted cysteine-rich proteins presumably localised in the fungal cell walls and contributing to the virulence of e.g. *Fusarium graminearum*, a necrotrophic fungal pathogen causing Fusarium head blight (FHB) disease of wheat, barley and other cereal grains. The knock-out mutant strains were more affected by treatments with chitinase and β-1,3-glucanase. The virulence of the mutants on wheat and soybean was not affected, thus indicating that cerato-platanin could protect fungal cell wall polysaccharides from enzymatic degradation (Quarantin et al. 2016).

Moreover, *Parastagonospora nodorum* necrotrophic effector SnTox1 interacted with a receptor on the outside of the plant cell to induce programmed cell death to acquire nutrients and the upregulation of PR genes including chitinases. Additionally, SnTox1 has structural homology to several plant chitin-binding proteins. Therefore, SnTox1 protected the different fungi from chitinase degradation (Liu et al. 2016).

VdCP1, a secreted *Verticillum dahliae* protein, is a conserved secretory protein, identified as a member of the SnodProt1 phytotoxin family. VdCP1 conferred resistance to *Botrytis cinerea* and *Pseudomonas syringae pv. tabaci* in tobacco and to *V. dahliae* in cotton. Further research revealed that VdCP1 possesses chitin-binding properties and that the growth of *vdcp1* knockout mutants was more affected by treatments with chitinase, indicating that VdCP1 could protect *V. dahliae* cell wall from enzymatic degradation (Zhang et al. 2017).

Fungal pathogens also secrete effectors that directly target host chitinases to inhibit their activity. Furthermore, fungi also have LysM effectors that prevent the recognition of released chitin oligomers by plants, which can be attributed to two mechanisms. Due to their ultrahigh affinity chitin binding, these effectors may either scavenge chitin fragments. Therefore, they avoid the activation of the host chitin receptors. Moreover, they prevent the chitin-induced receptor dimerisation and the activation of the chitin receptor complex (Sánchez-Vallet et al. 2015).

Avirulence (Avr) and necrotrophic effectors, also known as host-selective toxins, are the primary classes of host-specific effectors. Avr effectors are typically (but not exclusively) associated with biotrophic pathogens where R proteins conduct recognition. Plant NBS-LRR-containing R genes are involved in multiple layers of defence mechanisms as can accurately recognise and interact with corresponding pathogen *avr* genes (Yu et al. 2014).

*Cladosporium fulvum* (syn. *Passalora fulva*) causes leaf mould of tomato (*Solanum lycopersicum*) (Joosten and De Wit 1989; Thomma et al. 2005). In *C. fulvum* mutants deleted in *CfWor1*, which is a global transcriptional regulator (Okmen et al. 2014), expression of *Avr2, Avr4, Ecp6* genes were all highly reduced, particularly during the early stage of infection. Avr2 is an inhibitor of several plant Cys proteases that are required for a basal response but is recognised by the extracellular Cf-2 immune receptor (Rooney et al. 2005; Van Esse et al. 2008). Avr4 is a chitin-binding protein that protects the fungus from plant chitinases, and it is recognised by the *Hcr9-4D* LRR-RLP gene located at the *Cf-4* locus (Thomas et al. 1997; Van Den Burg et al. 2006). Ecp6, a LysM protein, also binds to chitin oligomers released by the action of host chitinases, which in turn minimises recognition by the host (Sánchez-Vallet et al. 2013). Reduced effector expression was proposed to contribute to the reduced virulence of *cfwor1* mutants (Okmen et al. 2014).

Avr4 homologs have also been identified in several other fungal species, including *Mycosphaerella fijiensis* (Stergiopoulos et al. 2010) and *Dothistroma septosporum* (De Wit et al. 2012). However, functional homologs of Ecp6 were found in *Zymoseptoria tritici* (Mg3LysM) and *Magnaporthe oryzae* (Slp1) (De Jonge et al. 2010; Marshall et al. 2011; Mentlak et al. 2012). Interestingly, the Mg3LysM effector of *Z. tritici* unites the abilities of both Avr4 and Ecp6 to sequester chitin oligomers and protect fungal cells from hydrolysis (Marshall et al. 2011).

Stergiopoulos et al. (2010), based on their work in *M. fijiensis* (Black Sigatoka, the most significant disease of bananas and plantains), proposed a function for Ecp2 (functional homolog of Ecp6) and Avr4. In the absence of Cf resistance proteins, Ecp2 promoted virulence by interacting with an *in-planta* target, causing host cell necrosis that facilitates the necrotrophic mode of nutrition of hemibiotrophs, such as *M. fijiensis*. In the case of biotrophs, such as *C. fulvum*, coevolution between host and pathogen was suggested to result in a fine-tuning of Ecp2, which only weakly perturb the host cells without inducing cell necrosis. In the presence of related Cf resistance proteins, Cf-Ecp2 guards the virulence target of Ecp2 and triggers a Cf-Ecp2-mediated hypersensitive response (HR) that is epistatic over virulence target-mediated necrosis. In contrast, Cf-4 presumably interacts directly with Avr4 and triggers an HR. Transfer of these Cf genes to plant species that are attacked by fungi containing these cognate core effectors provides unique ways for breeding disease-resistant crops.

Cell wall remodelling of fungal pathogens leads to reduced access of chitinases to the chitin in the cell wall. Several fungi accumulate indigestible α-1,3-glucan at the surface of the cell wall to prevent degradation of chitin by chitinases. For example, the α-1,3-glucan synthase gene MgAGS1 was not essential for infectious structure development but infection in *Magnaporthe oryzae*. Lack or degradation of surface α-1,3-glucan increased fungal susceptibility towards chitinase, suggesting the protective role of α-1,3-glucan against plants’ antifungal enzymes during infection (Fujikawa et al. 2012). Alternatively, during host colonisation, some fungi convert cell wall chitin by deaminases into chitosan, which is a poor substrate for chitinases and a weak inducer of plant immune responses (El Gueddari et al. 2002).

Hydrolytic elimination of plant chitinases also a possibility to avoid the “recognition” of the pathogen. In germinating maize embryos, as in response to infection by the fungus *Fusarium moniliforme*, induction of two acidic chitinase isozymes (Cordero et al. 1994) was shown. A 29,000 Da chitinase isolated from mature seeds of the *A*. *flavus*-resistant line Tex6 inhibited the growth of *A. flavus* (Moore et al. 2004). Although the exact identity of this chitinase was not specified, it was highly similar to the homologous chitinases A and B (Hawkins et al. 2015). Commercial maize hybrids have been shown to produce two different forms of the ChitA and the Chit B chitinase proteins, due to either difference in the genetic sequences or post-translational modifications (Naumann et al. 2009; Naumann and Wicklow 2010). Both forms of ChitA and ChitB appeared to be modified by proteases from the fungi *Bipolaris zeicola* (Naumann et al. 2009), *Stenocarpella maydis* (Naumann and Wicklow 2010) and *Fusarium verticillioides* (Naumann et al. 2011), which led to a reduction of plant chitinase function and allowed the fungi to overcome host barriers (Naumann et al. 2009, 2011; Naumann and Wicklow 2010).

*Colletotrichum graminicola* hemibiotrophic fungus causes maize anthracnose and produces a metalloprowtease (Cgfl) with a role in virulence. Transcriptional profiling experiments and live cell imaging showed that Cgfl, which is highly conserved in fungi, was explicitly expressed during the biotrophic stage of infection. *In vitro* chitinase activity assays of leaves infected with wild-type and null mutant strains show that, in the absence of Cgfl, maize leaves exhibit increased chitinase activity. Similarity searches, phylogenetic analysis, and transcriptional profiling revealed that *C. graminicola* encodes two LysM domain-containing homologs of Ecp6, suggesting that this fungus employs both Cgfl-mediated and LysM protein-mediated strategies to control chitin signalling (Sanz-Martín et al. 2016).

In barley, the parasitic fungus *Blumeria graminis f.sp. hordei* induces early upregulation of alcohol dehydrogenase 1 (ADH-1) activity in leaf epidermal tissue. Chitin-treatment induced systemic downregulation of ADH-1 activity and resistance to PM, while overexpression of ADH-1 inhibited the chitin-induced resistance to PM (Käsbauer et al. 2018).

## Biocontrol and other agricultural applications

Organic agriculture relies on employment of disease-resistant crop cultivars and techniques such as crop rotation, green manure, compost and biological disease control. Understanding the mechanism of plant–microbe interaction can facilitate and accelerate development of resistant cultivars and biological control. Plant–microbe contact can be roughly classified into two types, compatible and incompatible interactions, leading to the critical agronomic phenotypes of susceptibility and resistance to certain diseases, respectively. The incompatible interaction is extensively exploited by crop breeders to raise resistant cultivars for crop production in agriculture (Li et al. 2013).

Since the 1980s, crab and shrimp shell powder chitin and its deacetylation product chitosan have been used for crop farming as biopesticides, biofertilisers, and in seed coating formulation, and agricultural film (Ha and Huang 2007; El Hadrami et al. 2010; Trouvelot et al. 2014).

Chitinous materials control fungal diseases in plants by the indirect inhibition of the pathogens via their decomposition by-products, by also having a fertiliser effect, stimulating/supporting the growth of beneficial microorganisms, and by the elicitor activity of chitin (Velasquez and Pirela 2016). Various methods of application of chitosan and chitin are practised controlling or prevent the development of plant diseases or trigger plant innate defences against pathogens. The treatment has a long time effect as chitin amendment was found to raise the suppressiveness of soil for as much as two years following treatment. Moreover, during chitin amendment, microbial communities shifted in both the abundances and structures of both of total soil bacteria actinobacteria, *Oxalobacteriaceae* and fungi, in particular, *Verticillium dahliae* were recorded (Cretoiu et al. 2013). The richness of family-18 glycosyl hydrolase chitinase genes carried by the soil bacteria was also revealed in chitin- (Cretoiu et al. 2013) and chitosan-treated soil (Nampally et al. 2015). Chitin amendment in soil increases the vegetative growth of plants. Chitin tetramer oligosaccharide amendment was found to induce *Arabidopsis* genes that were principally related to vegetative growth, development, and carbon and nitrogen metabolism. Based on this finding, a low-molecular-weight chitin mix enriched to 92% with dimers, trimers and tetramers was produced for potential use in biotechnological processes. The low-molecular-weight chitin mix treated plants had increased *in vitro* fresh weight (10%), radicle length (25%) and total carbon and nitrogen content (6% and 8%, respectively) compared with untreated plants (Winkler et al. 2017).

Application of chitosan in biocontrol of plant pathogens has also been extensively explored, and the success depended on the pathosystem, the applied derivative, its concentration, degree of deacetylation, viscosity, and the used formulation (e.g. soil amendment, foliar application; chitosan alone or in association with other treatments). It is a nontoxic and biodegradable compound, as well as an elicitor. Therefore, it has the potential to become a new class of plant protectant, assisting towards the goal of sustainable agriculture (Bautista-Baños et al. 2006). In contrast to chitin, chitosan appears to elicit activity from plant cells via charge–charge interactions with negatively charged phospholipids instead of via a receptor-specific interaction (Kauss et al. 1989). The differential expression of key elements under SA and chitosan treatment were investigated by Coqueiro et al. (2015) in orange by RNA-seq technology. More genes were induced by SA treatment than by chitosan treatment. Under chitosan treatment, there were 640 differentially expressed genes, many of them involved in secondary metabolism and the treatment also altered some hormone metabolism pathways (Coqueiro et al. 2015). It was found that chitosan amendment increased plant phenylalanine ammonia lyase activity, which is the crucial enzyme of phenylpropanoid metabolism, and the subsequent increase in phenolic content, polyphenol oxidase, peroxidase, and chitinase is a general response associated with disease resistance (Anand et al. 2009; Mejía-Teniente et al. 2013). The use of oligo-chitosan with the degree of polymerisation of five significantly enhanced defensive activities of all four enzymes (Li et al. 2016). Chitosan was also found to increase the production of glucanohydrolases, specific phytoalexins with antifungal activity and of lignin-like material and reduced macerating enzymes such as polygalacturonases and pectin methylesterase (Ali et al. 2014). Chitosan amendment increased harvested yield for some horticultural and ornamental commodities, and also enhanced plant growth and suppressed some of the notorious soil-borne diseases even in soil-less production systems (*e.g*. Lafontaine and Benhamou 1996).

Control of the post-harvest diseases also important task of the biocontrol because it suggests alternatives to the use of pesticides on fresh produce in storage. The addition of chitosan stimulated degradation of pathogens in a way resembling the application of a hyperparasite (Benhamou 2004). Recent investigations on coating showed that chitosan extends the shelf life of treated fruit and vegetables owing to its ability to form a semipermeable coating minimise the rate of respiration and reduce water loss (Bautista-Baños et al. 2006). With chitosan coating, the ripening was delayed by modifying the internal atmosphere, which decreasing decays due to pathogens (El Ghaouth et al. 1992, 2000).

Several biopesticides or biofertilisers have been developed based on chito-oligosaccharides and chitosan-oligosaccharides. Interestingly, the level of the applications of these two oligosaccharides is very different because of the more developed production techniques of chitosan-oligosaccharides than those of chitooligosaccharides. The large-scale production technology of chitosan-oligosaccharides is highly developed and widely used; meanwhile, the mature chito-oligosaccharides production technology is lagging. Several commercial products have been developed and applied to agriculture. However, the method of application of these oligosaccharides is not optimised due to the lack of understanding of the related mechanisms (Yin et al. 2016). Therefore, the future task is to solve the large-scale production technology of chito-oligosaccharides; ensure the suitable degree of polymerisation and degree of acetylation-controlled production technology and elucidate the chitosan-oligosaccharides signal recognition and transduction in a controlled standardised environment in plants.
